# Treatment of patients with metastatic colorectal cancer and poor *performance status*: current evidence and challenges

**DOI:** 10.6061/clinics/2018/e542s

**Published:** 2018-09-17

**Authors:** Lucila Soares da Silva Rocha, Rachel P. Riechelmann

**Affiliations:** I Departamento de Radiologia e Oncologia, Instituto do Cancer do Estado de Sao Paulo (ICESP), Faculdade de Medicina FMUSP, Universidade de Sao Paulo, Sao Paulo, BR; II Departamento de Oncologia – AC Camargo Cancer Center, Sao Paulo, BR.; III Programa de Pós-Graduação em Oncologia, Faculdade de Medicina da Universidade de Sao Paulo, São Paulo, BR

**Keywords:** Metastatic Colorectal Cancer, Poor Performance, Chemotherapy, Performance, Survival

## Abstract

Patients with unresectable metastatic colorectal cancer live for a median of three years when treated with standard therapies. While the evidence guiding cancer-directed treatment of this disease comes from phase III trials that have mostly enrolled patients with good *performance status*, some patients present with poor clinical conditions. The best treatment for these patients remains to be determined. We performed a systematic review of the treatment outcomes of patients with metastatic colorectal cancer and poor *performance status,* defined as Eastern Cooperative Oncology Group performance status ≥2. Eligible articles were prospective or retrospective studies or case reports published in English, Portuguese or Spanish. We searched PubMed, EMBASE, LILACS and the Cochrane Library from onset until October 2017 using specific keywords for each search. We found a total of 18 publications, mostly case reports and retrospective studies (14 articles). One was an uncontrolled prospective trial, two were observational studies and one was an individual patient meta-analysis. Although some studies suggested benefits in terms of symptomatic response with standard chemotherapy, with good safety profiles when dose-reduced regimens were administered, a true survival gain could not be demonstrated. The scientific evidence for treating metastatic colorectal cancer patients with poor *performance status* is scarce, and more studies evaluating treatment for this population are necessary since this condition is not uncommon in clinical practice, particularly in the public healthcare system and developing countries and among destitute populations.

## INTRODUCTION

Nearly 50% of patients with colorectal cancer have metastatic or nonoperable disease at the time of diagnosis or will develop metastasis or a local recurrence in the following months to years 1. For patients with unresectable metastatic disease, the prognosis is bleak, with 5-year survival rates of 5% or less 2. Chemotherapy prolongs progression-free survival (PFS) and overall survival (OS) compared with best supportive care (BSC) alone 3 or polychemotherapy, specifically with FOLFOX (leucovorin + 5-fluorouracil (5-FU) + oxaliplatin) and FOLFIRI (leucovorin + 5-FU + irinotecan) regimens, increases the response rate compared with fluoropyrimidine monotherapy, and has become the standard first-line therapy for this disease. Bevacizumab (BEV) and anti-epidermal growth factor receptor (EGFR) monoclonal antibodies have also added survival benefits to chemotherapy alone; the latter is specifically useful in patients whose tumors are RAS wild-type. Currently, the median survival of patients with metastatic colorectal cancer (mCRC) can reach 30 months 4. However, the clinical benefits of various treatments are established in clinical trials that enroll highly selected patients with good *performance status* (PS), which is often measured using the Eastern Cooperative Oncology Group (ECOG) classification. Most patients included in mCRC trials present with ECOG PS 0-1, and only a minimal percentage of those with poor ECOG PS are enrolled. Therefore, there are scarce data to evaluate whether the benefits observed in clinical trials also apply to patients with poor PS. For example, in the landmark FOCUS trial, less than 10% of patients had ECOG PS 2 5.

Given the lack of robust evidence to guide treatment decisions for mCRC patients with poor ECOG PS, an individual patient data meta-analysis of randomized trials evaluated the outcomes of systemic chemotherapy in patients with ECOG PS 2, which represented 8% of the total sample 6. Patients with ECOG PS 2 experienced a doubled risk of grade 3/4 treatment-related toxicities and significantly worse outcomes, such as 60-day mortality rate (12% *vs.* 2.8%), lower response rates (43.8% *vs.* 32.0%), shorter median PFS (7.6 *vs.* 4.9 months) and shorter median OS (17.3 *vs.* 8.5 months) than those with ECOG PS 0-1. However, patients with even worse clinical conditions, such as those with ECOG PS 3-4 have not been represented at all in clinical trials. The results from a retrospective study performed by our group suggested that palliative chemotherapy might benefit mCRC patients with ECOG PS 3-4, as they experienced a low rate of serious adverse events and lived longer than those treated with BSC exclusively 7. However, those findings may reflect selection bias.

Therefore, we have performed a systematic review to assess the evidence for the treatment of mCRC patients with ECOG PS ≥2.

## MATERIALS AND METHODS

This was a systematic review of studies that evaluated the treatment outcomes of mCRC patients with poor ECOG/PS ≥2. Eligible publications were clinical trials, prospective or retrospective cohort studies, case-control studies, case reports or population-based studies and pooled analyses. We excluded editorials, commentaries, convention abstracts, literature reviews and articles written in other languages other than English, Portuguese or Spanish.

The PubMed, Embase, Cochrane Library and LILACS databases were searched using the following search terms: 1 ("Colorectal Neoplasms" [Mesh]) AND ((performance[Title] AND status[Title]) OR ECOG[Title]) in PubMed; 2 ('colorectal cancer'/exp OR 'colorectal neoplasms') AND ('chemotherapy'/exp OR chemotherapy) AND 'poor performance' in Embase (ScienceDirect); 3 (tw:("colorectal cancer")) AND (tw:("poor performance")) AND (tw:(chemotherapy)) in LILACS; and 4 “colorectal neoplasms” in the Cochrane Library. All searches were conducted from the inception of each database until October 31^st^, 2017. Additionally, the reference lists of all the retrieved articles were reviewed to identify additional eligible studies. This strategy was used because, after testing several combinations of key words, this was the search that retrieved the largest number of studies.

It was initially planned to have a third researcher independently extract the data from the studies. However, after selecting the eligible studies, the data were very heterogeneous, and the number of articles was quite small; thus, two investigators evaluated each article and discussed the contents, one investigator extracted the data, and then both discussed and revised all the data. For each eligible paper, the investigators collected information about study design, characteristics of the study population, type of cancer-directed therapy and percentage of poor PS patients and treatment outcomes, such as OS and treatment-related adverse events.

## RESULTS

**Figure 1:**
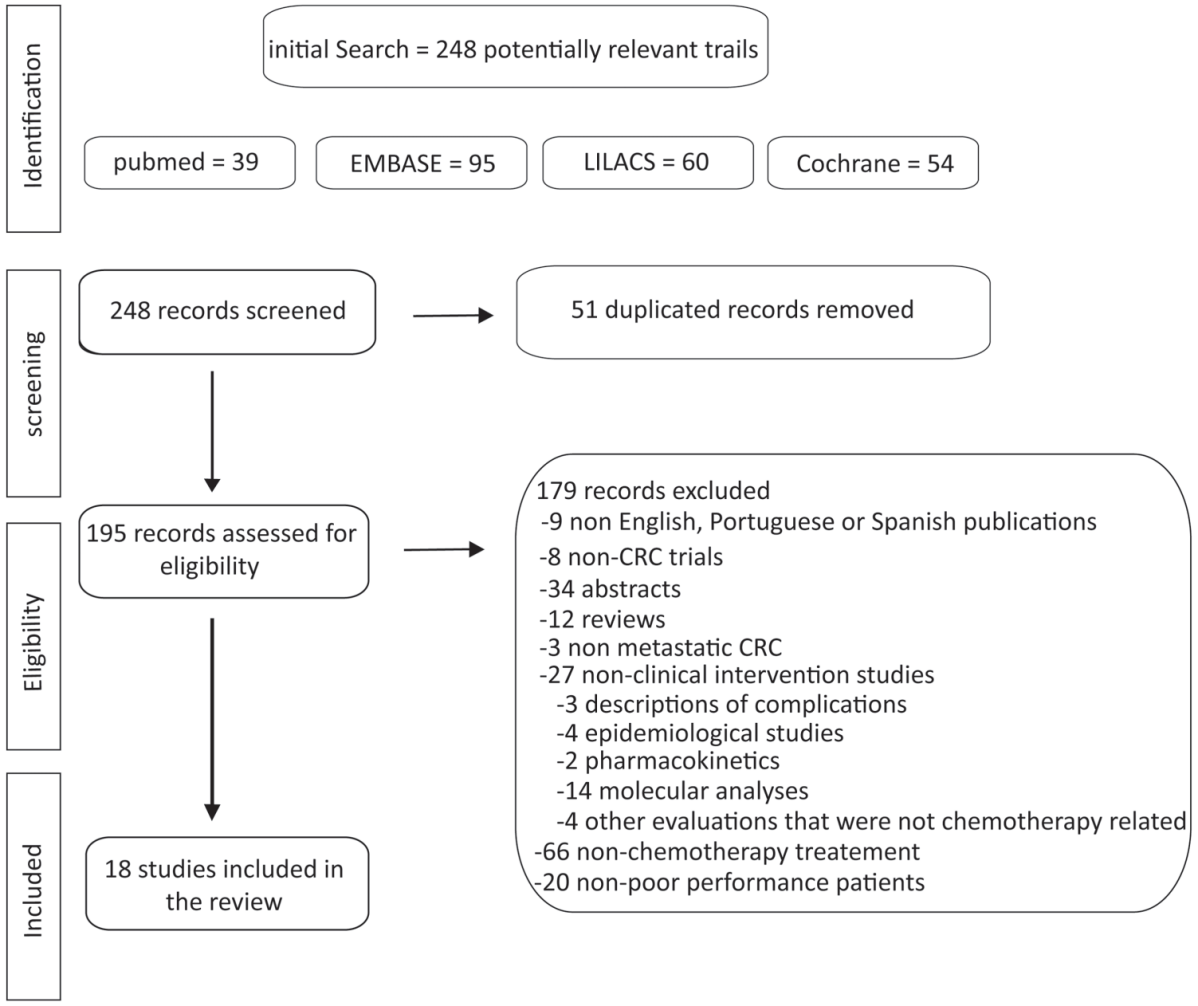
Flow chart of the search strategy and eligible articles.

Our initial search revealed 248 articles including 39 from PubMed, 95 from EMBASE, 60 from LILACS and 54 from the Cochrane Library, but only 18 were eligible. The number of studies evaluated and the sequential selection are presented in detail in Figure 1.

Of the selected articles, 11 were retrospective studies (61%), two were case series, two were uncontrolled prospective trials, two were observational studies and one was an individual patient meta-analysis. Table 1 summarizes the characteristics of the included studies.

Below we present the studies according to type of setting and population, as we tried to group the available evidence in a didactic form.

### Bevacizumab and anti-EGFR monoclonal antibodies

A case series of eight patients, most with ECOG PS 2 and one with ECOG PS 3, suggested that a combination of FOLFOX plus cetuximab for patients with RAS wild-type tumors who needed tumor shrinkage to alleviate symptoms related to extensive metastatic disease had clinical benefits. Improvement in ECOG PS, reduced jaundice and ascites, pain relief, resolution of intestinal obstruction and/or objective radiological response occurred in 75% of patients. Their median survival was 5.2 months (range, 2.5-14+ months) 8.

A retrospective study including 20.1% mCRC patients with ECOG PS ≥ 2, showed that patients with poorer PS and comorbidities were significantly more likely than those with better PS to have their tumors tested for RAS status at diagnosis in an attempt to induce tumor response with anti-EGFR monoclonal antibodies (until 60 days after diagnosis) 9. On the other hand, poor PS was one of the most common reasons for patients with KRAS wild-type tumors not to be treated with anti-EGFR antibodies 10.

Jehn et al. 11 performed a retrospective trial to investigate the combination of irinotecan plus cetuximab in 497 irinotecan-pretreated patients with KRAS wild-type tumors, of whom 22.9% had ECOG PS ≥2. A multivariate analysis evaluating the prognostic influence of age, PS, gender, age at diagnosis, primary location of mCRC, Charlson Comorbidity Index and skin toxicity showed that ECOG PS had a negative impact on PFS (hazard ratio (HR) = 0.61; 95% confidence interval (CI), 0.41–0.89; *P* = 0.01).

In a prospective phase II study of the combination of capecitabine + BEV, in which 62% of a total of 45 mCRC patients had ECOG ≥2, the overall median PFS was 6.87 months (95% CI, 5.1-11.5 months), the overall objective response rate (ORR) was 35%, and the median OS was 12.7 months (95% CI, 6.9-28.2 months); the most frequent grade 3/4 toxicities were diarrhea, fatigue and hand-foot syndrome 12.

Grande et al. 13 performed a retrospective study involving older and/or frail patients and showed that of 78% those who received first-line chemotherapy (31% received isolated 5-FU, 29% received oxaliplatin-based treatment, 35% received an irinotecan-based regimen, and 5% received other chemotherapy regimens), 14.6% presented with ECOG PS ≥2. Among them, those treated with chemotherapy experienced a longer 2-year OS rate (12.8% *vs.* 8.1%, *P* < 0.0001) than those treated with supportive care alone.

### First-line chemotherapy

A pooled analysis of nine clinical trials (including various chemotherapy regimens) with a total of 6,286 mCRC patients treated with first-line chemotherapy was performed of a subgroup of 509 (8%) patients with ECOG PS ≥2. For this subset, the analysis demonstrated that polychemotherapy led to improved outcomes compared with those of monotherapy in terms of PFS (HR = 0.78 for combination *vs.* monotherapy; 95% CI, 0.62 to 0.98; *P* = 0.0001), OS (HR = 0.79; 95% CI, 0.62 to 0.99; *P* = 0.04) and increased the likelihood of objective response (odds ratio (OR) = 2.85; 95% CI, 1.61 to 5.02; *P* = 0.0003). Patients with poor PS experienced lower response rates (OR = 0.61; *P* = 0.0001; 43.8% *vs.* 32%), shorter median PFS (HR = 1.52; *P* = 0.0001; median PFS, 7.6 *vs.* 4.9 months), and poorer OS (HR = 2.18; *P* = 0.0001; median OS, 17.3 *vs.* 8.5 months) than those with ECOG PS 0-1 and had an increased risk of grade 3 and 4 toxicities, particularly nausea (8.5% *vs.* 16.4%, *P* = 0.0001) and vomiting (7.6% *vs.* 11.9%, *P* = 0.006). Patients with ECOG PS 3-4 were not represented 6.

In a retrospective analysis of gastrointestinal cancer patients with poor PS (32 [27%] were mCRC patients) treated with different chemotherapy regimens, Shitara et al. 14 showed that the proportion of patients who experienced treatment-related mortality was low (0.8%). Despite their deteriorated health conditions, 10 (31.2%) mCRC patients had radiological and/or biochemical response (decrease in carcinoembryonic antigen (CEA) or carbohydrate antigen 19-9 (Ca19.9) levels ≥ 25% of baseline levels after 4 weeks of treatment) and/or clinical benefit defined by improvement in ECOG PS. For all responders (N = 78), they found a survival benefit compared with patients without tumor response (median OS, 6 *vs.* 2.2 months, *P* < 0.001).

Crosara et al. 7 performed a retrospective study of 140 patients with ECOG PS ≥ 2 (58.3% of the total sample) and found a median OS of 18.4 months for patients with ECOG PS 0-1, 10.8 months for those with ECOG PS 2 and 6.8 months for those with ECOG PS 3-4; all patients were treated with first-line oxaliplatin and/or 5-FU regimens. Although the findings were not statistically significant, this study still suggested that patients with ECOG PS 3-4 treated with chemotherapy presented prolonged median OS compared with those treated with BSC alone (6.8 *vs.* 2.3 months, respectively). They did not find differences in term of toxicities between the ECOG PS 3-4 group treated with chemotherapy *vs.* those who received BSC exclusively, but 40% of patients with ECOG PS 3-4 initially received dose-reduced chemotherapy.

A prospective observational trial analyzed the outcomes of mCRC patients who were included in clinical trials *vs.* those who were not, and 17% of the patients who were not included in clinical trials had poor PS. Of all patients, 36% were included in a clinical trial, and 32% received BSC alone; the most common reason for this treatment option was poor PS in 54% of patients, the presence of comorbidities in 14%, declination of treatment in 13%, very old age in 13% and other causes in 6%. Clinical trial patients presented prolonged OS (21.3 months) compared with patients treated with combination chemotherapy outside of a clinical trial (OS = 15.2 months) 15.

In a multivariate analysis performed as part of a retrospective study by Massacesi et al. 16, ECOG PS ≥ 2 (8.1% of the sample) was associated with short-term survival (defined as ≤ 6 months) and initial progression after first-line 5-FU-based chemotherapy regimens (OR = 3.42; 95% CI, 1.48-7.89; *P* = 0.015.

### Second- and subsequent-line therapy

Sørbye et al. 17 followed 112 patients after initial treatment with oxaliplatin-based regimens and observed that 47% of them did not receive second-line treatment with irinotecan-based chemotherapy. The main cause of this lack of second-line treatment was poor PS as defined by ECOG PS 2-4 in 59% of patients, which led to a median OS after first-line progression of 1.7 months.

In a retrospective trial involving 140 mCRC patients with ECOG ≥2 conducted by our group, thirty-seven (35%) received subsequent lines of chemotherapy, mostly based on irinotecan and fluoropyrimidine, suggesting that some patients derived sufficient benefit from first-line chemotherapy to be well enough to receive subsequent lines of treatment. Unfortunately, we were unable to evaluate the benefits of targeted anti-EGFR agents, which are reserved as a third-line treatment as per institutional protocol, because few patients with ECOG PS 3-4 lived long enough to receive third-line treatment 7.

A prospective trial conducted in two Spanish centers included patients with poor PS and tumors that were primarily resistant to 5-FU who received weekly second-line irinotecan. Considering the 26 mCRC patients with ECOG PS ≥ 2 (76.5% of the sample), 20.6% (95% CI, 6.3-34.9%) experienced tumor response, and 38.2% of patients had stable disease. The median OS was 8.3 months (95% CI, 1.7-1.69), and the most frequent grade 3/4 toxicities were neutropenia in 11 patients (32.4%) and diarrhea in 10 patients (29.4%) 18.

In another retrospective study of 25 patients, 8% had ECOG PS ≥ 2. Investigators evaluated the efficacy of IROX (irinotecan + oxaliplatin) in pretreated mCRC patients (median of 3 previous treatments [range, 2 to 7]), demonstrating an overall median OS of 7 months (95% CI, 6.2-7.8 months) and a disease control rate of 32%, without differentiating between patients with good *vs.* poor PS 19.

A case series including 4 patients (25%) with ECOG PS ≥ 2 treated with second-line irinotecan monotherapy demonstrated an overall partial response in 20% of patients and stable disease in 46.6%; 25% died within 30 days of beginning treatment 20.

### Hospitalized patients

In a retrospective trial of 199 inpatients with advanced incurable cancers of whom 18 (9%) had mCRC, palliative chemotherapy was administered in all the included patients, and 77% had improved symptoms and were discharged from hospital; 72% even received further chemotherapy, but their median survival was 4.5 months. This study did not present the results separately for mCRC patients 21.

## DISCUSSION

Even though some patients with mCRC present with a deteriorated PS in clinical practice, the best treatment for these patients remains unknown. In a previous retrospective study performed by our group, 27% of mCRC patients treated in a large public cancer center had ECOG PS 3-4 at the time the first-line treatment decision was made 7. Here, we present our findings of a systematic review of the literature to answer the pragmatic clinical question of how to treat these patients with poor PS. Despite the relevance of this topic, only a few studies, with most being retrospective cohort studies or case series, have evaluated the treatment outcomes of mCRC patients with poor ECOG PS. However, some studies have suggested treatment-associated benefits, such as radiological and/or symptomatic improvement, prolonged OS and low risk of grade 3/4 toxicities with dose-reduced therapies.

In 2012, the American Society of Clinical Oncology published their top five recommendations to improve cancer care and reduce costs, the first being “Do not use cancer-directed therapy for patients with solid tumors who have the following characteristics: low *performance status* (3 or 4), no benefit from prior evidence-based interventions, not eligible for a clinical trial, and with no strong evidence supporting the clinical value of further anticancer treatment” 22. However, we think the ECOG PS is a somewhat unrefined classification as it does not reflect the subtle aspects of clinical evaluation. For example, some patients without (or with a few) comorbid illnesses may have ECOG PS 3 or 4 that is exclusively induced by metastatic disease. In this scenario, tumor shrinkage may improve symptoms, for example, in patients with extensive bone metastases and/or medullary compression from a chemosensitive tumor. In this situation, oncologists must decide to either follow the evidence and/or expert guidelines that recommend BSC alone or risk treating patients without knowing the likelihood of treatment benefits. Additionally, patients may have poor PS due to chronic physical limitations or disabilities that do not necessarily pose risks to clinical trial participation, such as stable neurological sequelae from polio or stroke. Certainly, the treatment outcomes vary considerably in patients who are frail due oncologic disease *vs.* those who are frail because of comorbidities, severe organ dysfunction or advanced age, even though all these clinical conditions imply poor ECOG PS. Indeed, some studies included in this systematic review reported tumor shrinkage and even improvement in cancer-associated symptoms with standard chemotherapy. Therefore, we strongly advocate that the ECOG PS scale alone should not be used exclusively to guide treatment decisions. In these situations, other scales can help to make treatment decisions, such as the Edmonton Symptom Assessment System (ESAS) 23, which summarizes symptom burden, the Palliative Performance Scale 24, the prognostic Charlson Comorbidity Index 25, and scales that predict the probability of severe toxicities due to chemotherapy. 21,26 Last but not least, clinical judgment should prevail when the scientific evidence is lacking.

Even though combination chemotherapy improves response rates and, in some cases, disease-related symptoms compared with monochemotherapy in mCRC patients, those with poor PS have not been included in trials evaluating the combination of fluoropyrimidine with either oxaliplatin or irinotecan 27-31, and patients with ECOG PS ≥ 3 are systematically excluded from the main second- and third-line trials for CRC treatment 19,32-39. We have data from patients with good PS that shows that de-escalation of chemotherapy 40 or even complete cessation of chemotherapy does not compromise OS, while it preserves quality of ife 41. Additionally, the safety and benefits from adding BEV or anti-EGFR agents to standard chemotherapy in patients with poor ECOG PS are unknown because few patients with ECOG PS 2 and none with an ECOG PS 3-4 have been enrolled in phase III trials with these anticancer agents. Due to the paucity of evidence-based information to guide treatments for patients with poor PS, community oncologists make decisions based on clinical experience or intuition. Although reduction in dose intensity of treatment is a common alternative in clinical practice when dealing with frail patients, there are no data to demonstrate that reduced doses provide the same benefit as full dose regimens. Some authors have suggested that in the frailest patients, a lower dose of chemotherapy should be considered for the first cycle, and if this is well tolerated, a subsequent dose escalation may be tested 12. In our institutional retrospective study, patients with poor PS did not develop significantly more grade 3/4 toxicities probably because most of them were managed with initial dose reduction 7.

Additionally, because only 2-4% of patients with metastatic neoplasia are enrolled in clinical trials, which is much lower than the recommended rate of 10-15% 15, the overwhelming scientific evidence does not reflect the realities experienced by community oncologists. In that sense, trials that include relevant subsets of the cancer population must be conducted, *e.g.*, trials including patients with poor PS, elderly, HIV-positive individuals, and those with chronic kidney disease. In developing countries and in poor populations, the external validity of clinical trials may be even more problematic because late diagnosis and precarious access to a healthcare system often leads to patients presenting with more advanced disease, ultimately culminating in more patients with compromised PS at the time of presentation.

Forty percent of elderly patients with newly diagnosed colorectal cancer present with poor PS due to cancer complications or comorbidities 42. Consequently, data from studies with elderly patients are often extrapolated to nonelderly frail patients (ECOG PS ≥ 2) 12. Several studies involving elderly mCRC patients have demonstrated that fluoropyrimidine (5-FU or capecitabine) used in isolation or in combination with BEV provides prolonged median PFS, encouraging response rates and acceptable toxicities 12,43,44. While these findings provide the rationale for treating fragile patients with “more gentle” regimens, they should not be used alone to guide therapy in mCRC patients with poor ECOG PS.

Another important aspect that should be considered when deciding treatment strategies for mCRC patients with poor PS is the efficacy of the available therapies. While there is the generally accepted concept that performing chemotherapy during the last 30 days of a patient's life is not a good clinical practice 45, tumor subtype and chemosensitivity certainly influence patient outcomes and the indications for treatment. For example, a retrospective study showed that inpatients with clinical stage III ovarian cancer treated with platinum-based chemotherapy achieved median survival of 21 months 46. Additionally, with the modernization of cancer therapies and new drugs that lead to high response and low toxicity rates, oncologists should better evaluate the risk-benefit ratio of treating patients with poor PS, such as those whose tumors have DNA mismatch repair-deficient (dMMR)/microsatellite instability-high (MSI-H) and are very likely to benefit from immunotherapy 47,48.

A major limitation of the ability our systematic review to reach a conclusion on this topic was the small number of studies included and their limited quality. Additionally, because poor *PS* is sometimes evaluated as a secondary or even exploratory endpoints, we may have missed some studies in our search. Searching for Portuguese, English and Spanish articles may have led us to miss data from studies from developing countries where patients have limited access to healthcare and probably a high number of fragile patients are treated. Nevertheless, given the clear lack of research on mCRC patients with poor PS, we do not think that the inclusion of any unintentionally missed studies would have changed the overall picture of our systematic review.

In this systematic review we show that there are very few studies involving mCRC patients with poor PS, principally those with ECOG PS 3-4, which implies that these patients are systematically excluded from clinical trials. While some studies have suggested benefits in terms of symptomatic responses with standard chemotherapy, a true survival gain could not be demonstrated. Larger prospective epidemiological database studies are needed to elucidate whether and which patients with poor PS can benefit from treatment with palliative chemotherapy or from new targeted therapies and immunotherapy. Regarding clinical trials, we advocate that more flexible eligibility criteria be used and at least a limited number of patients with poor PS, especially those with poor ECOG PS and good organ function but with symptomatic burden, be included. Following the same logic, studies should separately analyze impaired PS due to active comorbidities, the sequelae of previous but stable disease and organ failure due to oncological disease.

## AUTHOR CONTRIBUTIONS

RP Riechelmann: concept and design, data selection, data interpretation, manuscript writing and final approval. Rocha L: data selection, data collection, data interpretation, manuscript writing and final approval.

## Figures and Tables

**Table 1:** Characteristics of studies included.

Reference	Patients (n)	ECOG PS ≥ 2% (n)	Study design	Chemotherapy protocol	MedianOS (months)	Results / conclusions
Nikolic-Tomasevic, et al. 2000 (20)	16	25 (4)	Retrospective	Second-line irinotecan	NR	Partial response, 20%; stable disease, 46.6%
Massacesi, et al. 2002 (16)	321	8 (26)	Retrospective	Various	15	Right and transverse colon primary tumors, younger age, ECOG PS ≥ 2, elevated CEA ≥ 5 g/l, site of metastatic disease and progression to first-line CT were associated with short-term survival; the median PFS in all treated in first-line CT = 5 m.
Benavides, et al. 2004 (18)	34	76.5 (26)	Phase II, single-arm	Second- or subsequent-line weekly irinotecan	8.3	Time to disease progression, 5.5 m (range, 0.9-17.5 m)
Sørbye, et al. 2007 (17)	112	14 (15)	Retrospective	FLIRI, FOLFIRI, CAPIRI	First-line - 8.9 Second-line - 20.8	More chance to receive second-line CT in ECOG PS < 2 (OR = 7.5) and alkaline phosphatase < 300 IU/L (OR = 2.5); OS = 1.7 m pts did not receive second-line irinotecan due poor PS; OS = 9.5 m and PFS = 4.1 m after beginning second-line irinotecan-based CT.
Shitara, et al. 2008 (14)	116	27.6 (32)	Retrospective	Various	2.2	32.7% of pts achieved a tumor response, decreased fluid accumulation (ascites and pleural effusion) or decreased in tumor markers; PS improvement = 13.8%
Nannini, et al. 2009 (49)	3	66 (2)	Case series	Metronomic capecitabine	NR	Treatment was well tolerated, and pts achieved long-term disease control for 6 and 15 cycles.
Sargent, et al. 2009 (6)	6286	8.09 (509)	Meta-analysis	Various	8.5	Median PFS = 7.6 *vs*. 4.9 months (ECOG PS < 2 *vs.* 2; HR = 1.52); grade > 2 nausea = 8.5% *vs*. 16.4% (ECOG PS < 2 *vs*. 2) and vomiting = 7.6% *vs.* 11.9% (ECOG PS < 2 *vs.* 2); 60-day all-cause mortality = 2.8% *vs.* 12.0% (ECOG PS < 2 *vs.* 2).
Sørbye, et al. 2009 (15)	760	17 (79)	Prospective observational	Various	15.8 (CT)2.8 (BSC)	36% of pts receiving CT were included in trials, and 32% received BSC alone.Trial-treated pts had a median OS of 21.3 m, and non-trial-treated pts had a median OS of 15.2 m; BSC was used because pts with poor PS had a median OS = 2.1 m.
Shitara, et al. 2010 (8)	8	87.5 (7)	Case series	FOLFOX + cetuximab	5.2	6 of 8 pts (75%) had clinical/radiological improvement.
Naeim, et al. 2013 (12)	45	62.2 (28)	Phase II, single-arm,	Capecitabine and bevacizumab	12.7	Median PFS = 6.87 m; ORR = 35% (16 pts) Grade 3-4 toxicities = 13.3-17.8%
Sgouros, et al. 2013 (19)	25	8 (2)	Retrospective	IROX	7	Disease control rate = 32%; median of 3 previous treatments (range, 2-7); median PFS = 3 m (95% CI, 2.3-3.7)
Jehn, et al. 2014 (11)	497	22.9 (114)	Retrospective	Cetuximab and irinotecan	NR	Median PFS = 5.9 *vs*. 6.1 m (Age < 65 *vs*. > 65 years) PS had a negative impact on PFS (HR = 0,499; 95% CI, 0.34-0.72)
Wheatley-Price, et al. 2014 (21)	199	9 (18)	Retrospective	Various	4.5	6 m survival rate = 41%; 77% of the treated inpatients were discharged home, and 72% received further CT.
Zheng, et al. 2014 (50)	7951	9.3 (742) * proxy for poor PS	Retrospective	5-FU + LV/IROX/IROX + biologics/Oxaliplatin + biologics		Pts with a proxy for poor performance were less likely to receive second-line treatment (HR = 0.82, *P* < 0.01)
Carter, et al. 2015 (9)	1363	20.1 (274)	Retrospective	NR		KRAS testing was more frequent if pts presented with lung metastases, poor PS, more comorbidities, and mCRC diagnosis after 2009.
Crosara Teixeira, et al. 2015 (7)	240	58.3 (140)	Retrospective	FLOX, FOLFOX, 5-FU	ECOG PS 2 = 10.8 ECOG PS 3-4 = 6.8	The median OS was longer in pts with ECOG PS > 2 treated with chemotherapy *vs.* those treated with BSC alone (6.8 *vs.* 2.3 months, *P* = 0.13).OS was shorter in pts with ECOG PS 2 than in those with ECOG PS 0-1 (HR = 1.67).
Grande, et al. 2016 (13)	751	14.6 (110)	Retrospective	Various	17	Not treated = median of 5 m Treated = median of 20 m
Ho, et al. 2016 (10)	443	??	Prospective observational	FOLFOX, FOLFIRI	22.3	The main reasons that KRAS wt pts did not receive anti-EGFR therapy were poor PS and death.

ECOG - Eastern Cooperative Oncology Group, BSC - best supportive care, CAPIRI - capecitabine + irinotecan, CEA - carcinoembryonic antigen, CT - chemotherapy, EGFR - epidermal growth factor receptor, FLIRI - 5-FU + irinotecan, FOLFIRI - 5-FU + irinotecan + oxaliplatin, FOLFOX - 5-FU + oxaliplatin, 5-FU - fluorouracil, HR - hazard ratio, IROX - irinotecan + oxaliplatin, m - months, n - number of patients, NR- not reported, OR - odds ratio, ORR - overall objective response rate, OS - overall survival, PFS - progression-free survival, PS - performance status, pts - patients, wt - wild-type. * proxy for poor performance - hospital bed use; oxygen use; walking aid use or wheel chair use.
